# A psychosocial risk factor model for female eating disorders: a European multicentre project

**DOI:** 10.1186/2050-2974-2-S1-O39

**Published:** 2014-11-24

**Authors:** Isabel Krug, Matthew Fuller-Tyszkiewicz, Sarah Mitchell, Fernando Fernandez-Aranda, Andreas Karwautz, Gudrun Wagner, Marija Anderluh, Laura Bellodi, Benedetta Nacmias, Valdo Rica, Sandro Sorbi, Kate Tchanturia, David Collier, Janet Treasure, Nadia Micali

**Affiliations:** 1 The University of Melbourne, Melbourne, Australia; 2 Deakin University, Melbourne, Australia; 3 Monash University, Melbourne, Australia; 4 University Hospital of Bellvitge, Barcelona, Spain; 5 University of Vienna, Vienna, Austria; 6 University Medical Centre Ljubljana, Ljubljana, Slovenia; 7 Fondazione Centro S.Raffaele del Monte Tabor, Milan, Italy; 8 University of Florence, Florence, Italy; 9 King's College London, London, UK; 10 University College London, London, UK

## Aim

To investigate through Structural Equation Modelling (SEM), the relationship between parenting styles, teasing about body weight/shape or eating, internalization of the thin ideal and body dissatisfaction amongst eating disorders (EDs) and controls and to see whether the risk factor model differed across various European countries and ED subtypes.

## Method

The total sample comprised 1373 participants(ED patients=618).The Cross-Cultural Questionnaire (CCQ) was used to assess the above-mentioned risk factors.

## Results

SEM analyses showed that the best fitting model was one allowing risk paths to vary across countries [χ2(425)=2105.271,p<.0001,RMSEA,=.022,CFI=.980]. In all countries teasing about weight/shape or eating was associated with body dissatisfaction(directly and via internalization of the thin ideal).There was a strong significant path from body dissatisfaction to ED(standardised path coefficient across countries:0.44-0.69, p<0.0001).Teasing about weight/shape or eating also directly predicted EDs (in the UK, Spain and Slovenia).There was however a weak effect of parenting on both teasing about weight/shape or eating and EDs directly.Risk models slightly varied across ED diagnoses.

## Conclusion

Our hypothesised model was partially confirmed; in particular the central role of teasing on EDs both directly and mediated by internalization of the thin ideal and body dissatisfaction was shown across five European countries.Conversely, the effect of parenting varied by country and therefore might have cross-cultural effects.

This abstract was presented in the **Prevention & Public Health** stream of the 2014 ANZAED Conference.

